# Changes in Occlusal Contacts upon the Cementation of Zirconia Crowns with Different Cement Spacers

**DOI:** 10.3390/dj12120377

**Published:** 2024-11-22

**Authors:** Yujun Wang, Philip Millstein, Korina Yun-Fan Lu, Jason D. Lee, Sang J. Lee

**Affiliations:** Department of Restorative Dentistry and Biomaterials Sciences, Harvard School of Dental Medicine, Boston, MA 02115, USA; yujun612@gmail.com (Y.W.); philip_millstein@hsdm.harvard.edu (P.M.); korina_lu@hsdm.harvard.edu (K.Y.-F.L.); jason_lee@hsdm.harvard.edu (J.D.L.)

**Keywords:** zirconia, cement space, occlusion, occlusal contact

## Abstract

****Background/Objectives**:** Occlusion plays a crucial role in the long-term success and functionality of dental restorations. The purpose of this study was to investigate the changes in occlusal contacts upon the cementation of zirconia crowns with different cement spacer settings in computer-aided design and computer-aided manufacturing (CAD-CAM) software (3Shape Dental System version 2.102.1.0). **Methods:** A master model of a prepared abutment for a crown on the right maxillary first molar was scanned, and 30 sets of sample casts and zirconia crowns were fabricated with varying cement spaces (70 μm and 120 μm). These casts were mounted in maximal intercuspation (MIP) on a semi-adjustable articulator. Pre-cementation adjustments were made to fit the crowns and maintain the existing occlusion. Occlusal records were taken before and after cementation using polyvinyl siloxane impression material. These records were analyzed using a DC light box and image analyzer to measure changes in contact area, intensity, and patterns. Paired sample t-tests were used to compare pre- and post-cementation occlusal contact areas of each sample (α = 0.05). **Results:** Significant differences in occlusal contact areas were found between pre- and post-cementation in both groups (*p* < 0.001). The mean post-cementation contact surface area for the 70 μm group was 6281 ± 3310 μm^2^, compared to 2339 ± 1206 μm^2^ before cementation. For the 120 μm group, the post-cementation area was 5545 ± 3491 μm^2^, compared to 2071 ± 909 μm^2^ before cementation. An increase in contact intensity was also observed after cementation. **Conclusions:** This study demonstrates that cementation increases occlusal contact surface area and intensity in both cement space groups.

## 1. Introduction

Occlusion plays a pivotal role in restorative dentistry, significantly influencing the mechanical stability, wear resistance, and overall longevity of dental restorations. A well-designed occlusion allows the restoration to integrate seamlessly into the stomatognathic system, ensuring the even distribution of forces across the teeth and minimizing internal stresses, wear, and the risk of failure. Misaligned occlusion can introduce static and dynamic interferences, potentially causing premature failure of the restoration [1,2]. Given the critical role of occlusion, post-cementation adjustments are often necessary to refine the occlusal alignment and optimize the restoration’s performance [3,4,5]. Factors that affect the occlusion of restorations include the accuracy of impressions, bite registration, and fabrication errors from casting or three-dimensional (3D) printing technologies, as well as cementation errors and marginal adaptation of the final restoration [6,7]. Studying occlusion presents significant challenges due to the intricacies of jaw movements, individual anatomical variations, and the dynamic forces involved in bite mechanics. Emerging tools, such as electronic tracking devices and optical jaw tracking systems, have improved precision in measuring and understanding occlusal forces [8]. In this study’s in-vitro setting, where individual patient factors such as muscular dynamics do not influence occlusal forces, we employed bite registration and a controlled light source to study occlusion. This approach enabled a standardized and quantifiable analysis of occlusal load before and after cementation, minimizing variability and thus enhancing the reliability of the experimental results [6].

In light of the importance of occlusion, technological advancements such as computer-aided design and computer-aided manufacturing (CAD-CAM) have emerged to enhance the restoration process significantly. CAD-CAM allows for the precise manipulation of design settings and adjustments, including restorative material thickness and cement space, during the virtual three-dimensional (3D) design of the restoration [9,10]. One of the notable advantages of CAD-CAM crowns over conventional ceramo-metal restorations is the ability to visualize the occlusal design and simulate both static esthetics and dynamic functionalities, providing a more comprehensive approach to dental care [11,12]. The integration of jaw motion tracking systems into the digital workflow also allows for more accurate simulation of a patient’s anatomical movement and precise identification of centric relation (CR), ensuring that the restoration not only fits well but also aligns harmoniously with the patient’s bite and appearance [11,12].

An important factor in ensuring the fit and longevity of a crown is the cement space, which refers to the small gap designed to accommodate the dental cement used to bond the crown to the tooth. In CAD-CAM systems, the cement space parameter is controlled by the technician and the dentist within the software, with a recommended minimum of 50 microns (μm) to achieve good marginal adaptation [13]. However, research has shown mixed results regarding the impact of cement space on restoration fit. Some research suggests that increasing the cement space setting of cast restorations can improve the fit of the restorations, [13,14,15,16,17], while other studies indicate that a smaller cement space improves the fit and retention [9,18]. These studies point out that optimizing cement spacer configuration to achieve a better marginal fit has a significant impact on reducing potential complications like marginal leakage and secondary caries [11,12,13,14,15,16]. Despite these varied findings, the specific effects of cement space on post-cementation occlusal changes in zirconia crowns remain unclear. Creating adequate cement space during CAD-CAM crown fabrication is crucial for ensuring a proper fit between the coping and the abutments, facilitating the removal of excess cement, and minimizing the force required to properly seat the crown during cementation [15]. Insufficient space can result in poor marginal adaptation, increased occlusal stress, and a higher risk of secondary caries [13,15].

The objective of this research project was to investigate whether and how different cement space settings in CAD-CAM crowns impact the occlusion upon cementation. Two cement space settings, 70 μm and 120 μm, were evaluated and compared in terms of their impact on occlusion. The null hypothesis for this study was that there would be no significant difference in contact surface areas between pre- and post-cementation in either group following the cementation of crowns with 70 μm and 120 μm cement space settings.

## 2. Materials and Methods

For the all-ceramic crown restorations, tooth preparation was carried out with the Brasseler diamond bur system (Brasseler, Savannah, GA, USA) and completed as horizontal preparation with a chamfer finish line. The master model (Columbia Dentoform Corp, Long Island, NY, USA) with the maxillary right first molar abutment prepared for an all-ceramic crown was scanned with a desktop scanner (E3; 3Shape A/S, Copenhagen, Denmark). The digital impression was used to print 30 identical sets of sample casts (Pro S Dental 3D Printer, Sprint Ray, Los Angeles, CA, USA). Each set of casts was mounted in maximum intercuspation (MIP) on a semi-adjustable articulator (Artex CR; Amann Girrbach AG, Maeder, Austria) with Type III gypsum (Mounting Stone, Whip Mix), by a single provider. A crown for the maxillary right first molar was designed in dental design software (3Shape Dental System version 2.102.1.0; 3shape, Copenhagen, Denmark) from the original scans of the master models and their bite registrations. Thirty zirconia crowns (Katana zirconia, Kuraray Noritake Dental, Tokyo, Japan) were fabricated using a milling unit (DWX-52D-5-Axis Dental Milling Machine; Roland DGA Corp, Irvine, CA, USA) based on the identical design with different virtual cement space along the axial walls. Fifteen sample crowns were fabricated with a 70 μm cement space, and the other fifteen were fabricated with a 120 μm cement space. The cement spacer was applied to all intaglio surfaces, including the marginal gap, of the crown. Each sample crown was randomly assigned to a set of mounted casts.

**Figure 1:**
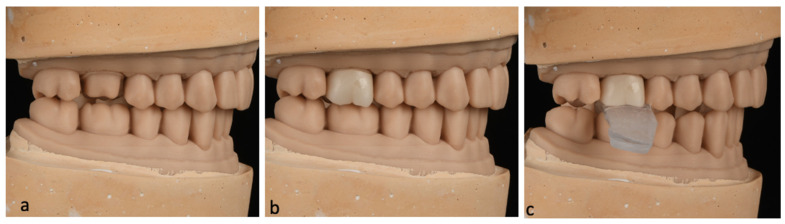
Cementation of the sample. (**a**) Mounted Casts, (**b**) sample try-in, (**c**) cementation with an insertion guide.

Prior to the cementation of each sample, adjustments in the interproximal and occlusal contacts were made to confirm the fit and seat of the crown on the cast and maintain the existing occlusion at MIP. Following the adjustments, a crown insertion guide was made for each sample using thermoplastic material that was heat-molded onto the occlusal surface while the crown was held under a 5 kg static load to ensure correct insertion orientation during cementation and simulate the insertion process in a clinical setting (Figure 1).

In order to evaluate and compare the occlusal contacts upon the cementation, two occlusal records were taken for each sample: a pre-cementation and a post-cementation occlusal record. A pre-cementation occlusal record was obtained for each sample using polyvinyl siloxane (PVS) (Regisil 2X, Dentsply Sirona, Charlotte, NC, USA) with a triple tray on the articulator under a 5 kg static load. After taking the pre-cementation record, the sample was then cemented with Self-Adhesive Universal Resin Cement (Relyx Unicem, 3M, Maplewood, MN, USA) following manufacturer instructions. Cementation was performed on the articulator under a 5 kg static load with the insertion guide in place. For each sample, a post-cementation occlusal record was taken in the same manner as the pre-cementation record.

Measurements were taken with a imagine process station, which is composed of a calibrated direct current lightbox (Schott North America, Elmsford, NY, USA), a grayscale digital camera (CFW-1308M, Scion Corporation, Frederick, MD, USA), and a computer with an imaging analysis program (ImageJ software version 1.54, National Institutes of Health, Bethesda, MD, USA). Pre- and post-cementation occlusal records of each sample were photographed on the light box and converted into digital images.

**Figure 2:**
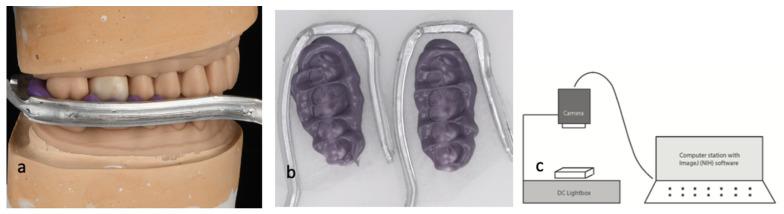
Occlusal record scan. (**a**) Cementation record taking, (**b**) PVS records scan, (**c**) DC light box set-up.

During scanning, the DC light box provided uniform lighting across the entire PVS record, allowing varying amounts of light to pass through according to the thickness of the material, which was relayed to the scan. The pre- and post-cementation records of each sample were scanned and converted into a light intensity map (Figure 2). The study focused on testing the sample crowns, recording and documenting two key changes, including occlusal contact area and contact intensity after cementation.

The contact area (in μm^2^) of each scan was calculated by selecting the areas with the highest light intensity on each scan within the occlusal table of the testing tooth. Changes in contact surface area (in μm^2^) between pre- and post-cementation were documented for each sample. Within each group, we used the changed surface area value of each sample to study if a significant increase in contact surface area was induced by cementation. To determine whether cementation changed occlusion, paired sample t-tests were used to compare the pre- and post-cementation occlusal contact areas of each sample (α = 0.05). Shifts in occlusal table positioning were not evaluated since pre- and post-evaluations could not be compared.

Changes in contact intensity were determined by measuring and comparing the highest light intensity value (in grayscale values) of each sample’s pre- and post-cementation records. As discussed previously, the amount of light passing through the scan correlated with the thickness of the testing material. An increased light intensity reading indicated an increase in occlusion intensity. The proportions of samples exhibiting an intensity increase were documented and compared between the two groups.

## 3. Results

### 3.1. Contact Surface Area

**Figure 3:**
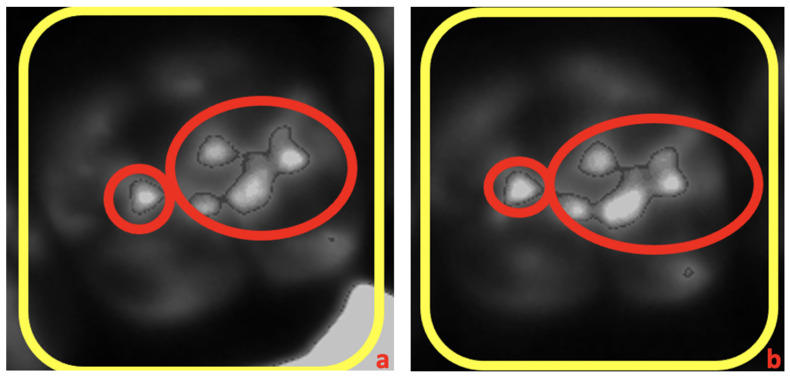
Output from occlusal record scan. Red circles show the occlusal contacts. (**a**) Pre-cementation scan, (**b**) post cementation scan.

Areas of occlusal contacts from the occlusal record scan at the time of pre- and post-cementation measurements are indicated by red circles (Figure 3). The means and standard deviations of the occlusal contact areas before and after cementation of the 70 μm group and 120 μm group are listed in Table 1.

In the 70 μm group, the pre-cementation contact area was 2339 ± 1206 μm^2^ (mean ± standard deviation), while the post-cementation measurement was 6281 ± 3310 μm^2^. The post-cementation contact surface area demonstrated a significant increase compared with the pre-cementation area (*p* < 0.001).

The post-cementation of the 120 μm group also had a significant increase, with a mean contact area of 5545 μm^2^ and a standard deviation of 3491 μm^2^ compared with 2071 ± 909 μm^2^ before cementation (*p* < 0.001).

### 3.2. Occlusal Intensity Changes

The changes in occlusal intensity were determined by measuring and comparing the light intensity (in grayscale values) of each sample’s pre- and post-cementation records.

The 70 μm group demonstrated an intensity increase in grayscale value of 0–10 in 47% of the samples, an increase in grayscale value of 10–20 in 7% of the samples, and an increase in grayscale value of 20–30 in 20% of the samples (Table 2). The 120 μm group demonstrated intensity increases in grayscale value of 0–10, 10–20, and 20–30 in 33%, 17%, and 7% of the samples, respectively (Table 2). The remaining samples did not show significant changes in light intensity levels.

## 4. Discussion

The impact of dental occlusion on various aspects of dental treatment is well recognized [2]. Occlusion plays a critical role in the success and longevity of dental restorations, as well as in patient comfort and functional efficiency [2]. Proper occlusal alignment ensures that the forces exerted during mastication are evenly distributed, which helps prevent damage to the dental prosthesis and surrounding structures [2]. Misalignment, on the other hand, can lead to complications such as tooth wear, temporomandibular joint disorders, muscle dysfunctions, and periodontal issues [2]. Ensuring proper comfort and function often necessitates occlusal adjustments to dental prostheses following cementation [3,4]. However, these adjustments can introduce issues such as increased chairside time, loss of crown anatomy, reduced material strength, and increased surface roughness [19,20]. This study aimed to investigate the effect of cement space on occlusion upon the cementation.

Various methods have been used to study occlusion. Traditional techniques include the use of articulating paper, which marks occlusal contacts by leaving ink impressions on teeth surfaces, yet it is limited by subjectivity in interpretation. Digital methods like Tekscan’s computerized occlusal analysis offer real-time data on bite forces, timing, and contact points through pressure-sensitive sensors [21]. In our current methodology, occlusal records were analyzed using bite registration, a lightbox, and imaging software to visualize and quantify occlusal areas and evaluate occlusal intensity with high precision and sensitivity [22].

Significant increases in the occlusal contact areas were observed in both the 70 μm group and the 120 μm group. The mean post-cementation occlusal contact areas more than doubled compared with the pre-cementation area in both groups. This finding indicates that cementation of indirect restorations significantly alters occlusion, leading to the rejection of the null hypothesis. Cementation induced a coronal shift of the restorations, which brought more area into occlusion. Consequently, the position of the crown was no longer consistent with the pre-cementation position, necessitating occlusal adjustments in a clinical setting.

This study also found that contact intensity and occlusal contact patterns were altered upon cementation. The seating of each crown prior to cementation exhibited numerous positional areas for seating crowns due to loose fit depending on the amount of cement space. Many samples in both groups demonstrated a shift from point contact to area contact after cementation. In extreme cases, some samples had contact points that shifted from the main functional areas, like the central fossa, to the inclines of cusps. This occlusal contact pattern shift can induce instability in occlusion and may lead to premature wear, microfractures, or even restoration failure if not properly adjusted [1,2]. It was difficult to assess the findings because the larger inherent cement space overshadowed the planned cement spaces. Consequently, the standard deviations in both groups were amplified.

The bite registration records were digitized to facilitate analysis using the DC lightbox and occlusion analysis software. In terms of contact intensity, variations in the thickness of the registration material across different contact areas were presented by the light intensity. The thinner registration material at solid contact areas allowed more light to pass through, which was then captured by the overhead camera. The light intensity measurement was directly proportional to the contact intensity. The majority of samples in both groups showed an increase in contact intensity following cementation, as documented by the changes in grayscale pixel values. The 70 μm group had a higher proportion of samples with increased intensity compared with the 120 μm group. An increase in contact intensity indicates a hyper-occlusion in the area and may induce adverse effects, such as occlusal trauma, if it is not adjusted properly [4]. For example, the intensity of occlusion is crucial in cases of cement-retained implant restorations, since implants lack periodontal ligaments that function as shock absorbers [23].

Combining all findings, the present study demonstrated that the cementation of full coverage restorations in both the 70 μm and 120 μm cement spacer groups altered the occlusion. The smaller cement space group has a higher chance of encountering situations like hyper-occlusion and shifting of contact areas. Therefore, it is strongly recommended to perform a thorough occlusal evaluation following cementation. It is important to note that post-cementation grinding of cemented crowns can result in a fragile crown with a roughened surface. Proper planning and surface finishing are important aspects of maintaining treatment success.

The limitations of this study include its in vitro design using typodont models, which did not capture the role of mastication muscles and TMJ. Additionally, the insertion guide method used in this study is less commonly used in clinical practice than simpler methods like cotton roll insertion, which may affect the generalizability of the findings. Incorporating adjacent teeth as a control could make this study more informative and complete. Future research could build on this study to investigate the optimal cement space setting for digital crown fabrication [24,25]. The use of a bite registration scan armamentarium enabled the documentation and visualization of occlusion, which could be an invaluable tool for practicing clinicians and for long-term patient follow-up.

## 5. Conclusions

This study demonstrated significant increases in occlusal contact areas and physical movement of the occlusal table when comparing post- to pre-cementation contact areas for different cement space groups. Notably, changes in contact areas and occlusal intensity following cementation were observed in both the 70 μm and 120 μm cement spacer groups. Cementation with a large cement space may translate into a physical shift in the prescribed occlusion and a smaller cement space result in hyperocclusion. Using a combination of bite registration, a controlled light source, and imaging analysis software, our methodology enabled precise visualizations of occlusal changes before and after cementation. These findings highlight the critical importance of conducting thorough occlusal evaluations after cementation, ensuring optimal function and enhancing patient comfort. Future research could also incorporate patient-specific factors by employing advanced tracking technologies, such as electronic and optical jaw tracking devices, which offer dynamic insights into mandibular movements and occlusal interactions under clinical conditions.

## Figures and Tables

**Table 1:** Mean pre- and post-cementation contact surface area (μm^2^) ± standard deviation (μm^2^) in the 70 μm and the 120 μm group.

Group	Pre-Cementation (μm^2^, Mean ± SD ^1^)	Post-Cementation (μm^2^, Mean ± SD)
70 μm	2339 ± 1206	6281 ± 3310
120 μm	2071 ± 909	5545 ± 3491

^1^ SD: standard deviation.

**Table 2:** Occlusal intensity changes in grayscale values after cementation between the 70 μm group and the 120 μm group. Percentage of samples with three levels of intensity changes are shown.

Group	Intensity Increase of 0–10 Grayscale Value	Intensity Increase of 10–20 Grayscale Value	Intensity Increase of 20–30 Grayscale Value
70 μm	47%	7%	20%
120 μm	33%	17%	7%

## Data Availability

The data presented in this study are available on request from the corresponding author.
